# The Role of Supportive Food Environments to Enable Healthier Choices When Eating Meals Prepared Outside the Home: Findings from Focus Groups of 18 to 30-Year-Olds

**DOI:** 10.3390/nu11092217

**Published:** 2019-09-13

**Authors:** Margaret Allman-Farinelli, Hassan Rahman, Monica Nour, Lyndal Wellard-Cole, Wendy L. Watson

**Affiliations:** 1 The University of Sydney, Nutrition and Dietetics Group, Charles Perkins Centre, School of Life and Environmental Sciences, The University of Sydney, Sydney, NSW 2006, Australia; hrah9994@uni.sydney.edu.au (H.R.); monica.nour@sydney.edu.au (M.N.); lyndalw@nswcc.org.au (L.W.-C.); 2 Cancer Prevention and Advocacy Division, Cancer Council NSW, Sydney, NSW 2011, Australia; wendyw@nswcc.org.au

**Keywords:** take-away food, fast food, restaurant, menu-labelling, food costs, healthy eating, young adult, food environment, smartphone

## Abstract

Young adults are the highest consumers of food prepared outside home, which has been linked to weight gain. The aim of this qualitative research was to gather opinions from young adults about what influences their food choice when eating out and if they so desired, what might enable healthy choices. Thirty-one adults aged 18 to 30 years participated in four focus groups (females = 24). Predetermined questions were used to guide discussions which were audiotaped then transcribed. The content was organized into themes and sub-themes using NVivo software. Two broad groupings arose—personal behaviors and changes to physical and social food environments. For many, eating out was viewed as a special occasion so that healthy food was not a priority and despite understanding health consequences of poor diets this was not an immediate concern. Price discounts and menu-labelling were suggested and trust in credible organizations and peers’ endorsement of meals and venues expressed. The role of smartphones in the modern food environment emerged as a tool to enable immediate access to many restaurants to order food and access reviews and social media as a source of persuasive food imagery. Current menu-labelling initiatives should continue, food pricing be explored and influence of smartphones on diet further researched. However, these findings may be limited by the high proportion of women and higher socioeconomic status and urban residence of many participants.

## 1. Introduction

The rate of weight gain today’s young adults are experiencing is greater than that by previous generations in many countries [1,2]. Diet is a major contributor to weight gain and the importance of understanding eating habits is evident. Young adults generally have poor quality diets in countries like Australia and the US [3]. Unfavorable dietary behaviors in young adulthood are especially concerning as these may influence both short- and long-term diet-related health outcomes [1].

A systematic review by Lachat et al. reported an association between ‘eating out-of-home’ and higher total energy intake [4]. Similarly, Summerbell et al. identified four prospective cohort studies in adults that found consumption of ‘fast food’ was associated with subsequent excess weight gain, obesity and type 2 diabetes [5]. Specifically, in young adults the US Coronary Artery Risk Development in Young Adults study (CARDIA) found that the mean weight gain over 15 years in those who ate at a fast food outlet less than once per week was 4.5 kg less than for those who ate at them more than twice per week [6]. Thus, food prepared away from home may be a significant contributor to weight gain in young adults.

In the past 40 years multinational take-away food chains have expanded rapidly [7], and this coupled with time limitations has driven the rise of the fast food culture. Young adults are the most frequent consumers of fast foods and subsequently spend the largest proportion of their food budget on fast foods and eating out [8]. The food choices of young adults are influenced by multiple factors, including life stage changes such as moving out of the parental family home, limited food budgets, low levels of cooking literacy and poor time management skills. Foods which taste good, are convenient and are low cost are all prioritized [9]. This positions young adults as a highly desirable target population for energy-dense, nutrient poor food and beverage marketing. Currently, the level of advocacy to improve young adults’ diets is low, nutrition promotion for them is limited and no measures have been introduced to regulate food advertising to this demographic [10].

Our recent quantitative study in 1001 18–30-year-olds conducted across NSW, Australia measured their take-away food habits [11]. Results suggested that 25% of meals and snacks were prepared outside the home (unpublished) but they provided 40% of energy and 36–45% of nutrients of public health concern such as sodium, sugars and saturated fat. Reducing take-away food consumption and/or supporting young adults to make healthier choices when eating out of home should be a priority for public health and should form part of obesity prevention initiatives. However, to design any program or policy a deeper understanding of young adults’ opinions on the types of messages and environmental changes that would encourage them to select healthier options when eating restaurant and take-away meals is required.

Thus, the aim of this study was to gather opinions of young adults (18–30 years-old) on the factors that influence their food choices when eating food prepared outside the home; types of messages that would encourage them to select healthy meal choices when eating out; and their views on developing more supportive food environments for healthier eating.

## 2. Materials and Methods

### 2.1. Study Design

Young adults aged between 18 and 30 years-old living in the greater Sydney area NSW, Australia, were recruited to participate in a one-hour focus group to openly discuss opinions regarding the described “research question”—factors influencing food selections when eating outside home, the role, if any, of health factors, and suggestions for what would influence them to select the healthy options. The methods of Krueger and Casey were used to develop and conduct the focus groups [12]. A facilitator prompt sheet was developed to guide the group discussion. Focus groups were held in meeting rooms in a range of locations (central and suburban campus of a large university with 80,000 staff and students) to enable recruitment of students and workers from a range of socioeconomic groups. The intent was to recruit approximately 32 to 40 young adults to focus groups with participant size 8 to 12 per group. A sample size of 25–35 is generally accepted within the literature as sufficient to reach information saturation [13]. The institutional Human Ethics Research Committee approved the study and all participants received information about the study and lead researchers and gave consent.

### 2.2. Participants and Recruitment

A range of methods were used to recruit potential participants and included: social media—an advertisement for the study was sent to existing friends, connections, posted on social media pages of Cancer Council NSW and paid advertising on Facebook; flyers posted on public notice boards on University campuses; and advertisements in electronic newsletters, mailing lists and web pages of the university and/or Cancer Council NSW. Participants contacted the research team online and were directed to a screener to assess their eligibility for the study.

Individuals had to fulfil the following selection criteria: aged 18 to 30 years-old and not pregnant or breast-feeding, and eating out at least twice per week. Exclusion criteria were being diagnosed or treated for the following medical conditions Anorexia Nervosa, Bulimia Nervosa or other eating disorder.

Participants received a $30 voucher as a reimbursement after completing the focus group. This offer should not impact the voluntary nature of consent as it is given after completion of the focus group rather than at the time of consent.

### 2.3. Screening and Data Collection

Basic demographic information was collected through the screening survey. This included name, age, gender, employment status, living situation, income, postcode, and level of education. The survey was hosted on Research Electronic Data Capture (REDCAP), a secure platform [14]. Data downloaded from the survey was stored on the university drive and only accessible through the researchers’ password-protected laptop.

### 2.4. Procedure

The 60-min focus groups were led by a female dietitian facilitator (APD PhD) experienced in conducting focus groups (MN) and assisted by a research student dietitian (HR). Other nutritionist observers attended the focus groups on a rotating basis (WW LWC). The facilitator guided the focus group discussion using pre-determined questions and prompts to allow for open discussion within the group, whilst ensuring key research questions were addressed (see Table 1). The focus groups took place between February and March 2019. All of the focus groups were audio recorded to ensure all feedback was captured.

### 2.5. Analysis

The recorded material from the four focus groups was transcribed verbatim and uploaded into NVivo software (version 11.0.0317 QSR International Pty Ltd., Melbourne, VIC, Australia). A deductive approach based on content was used to derive major themes and then sub-themes in the data. One researcher did the initial coding which was checked by a second coder (HR MAF). Quotes from the transcribed audio recording were de-identified to protect the participants. The consolidated criteria for reporting qualitative research (COREQ) checklist was used in the preparation and reporting of this qualitative study [15].

## 3. Results

A total of 105 young adults completed the on-line screener survey but only 31 attended focus groups (M = 7, F = 24). Groups 1 and 2 had seven participants each (2 males in each), group 3 had 8 participants (1 male) and group 4 had 9 participants (2 male) with an additional participant failing to attend focus group 4. Twenty-one participants were aged 18 to 24 years and ten 25 to 30 years. The majority were studying full or part-time with or without paid employment (*n* = 22), seven were in the workforce and two unemployed. Six participants were classified in the most disadvantaged quintiles for socioeconomic status, six in the third quintile and 19 in the highest quintiles.

The themes emerged around two distinct actionable areas. Firstly, personal behaviors and how these might be influenced and secondly, policy- or practice-relevant ideas for environmental change to enable young adults to change food-related behaviors. Under these major themes we further categorized the data into the sub-themes that were consistently raised during the four focus groups: (i) indulgence when eating out, (ii) health consequences of food and eating out, (iii) the smartphone as an influencer of food choice, (iv) food pricing, (v) menu labelling and (vi) endorsements of foods and outlets. Table 2 outlines the quotations that illustrate each sub-theme.

### 3.1. Personal Behaviors

#### 3.1.1. Indulgence When Eating Out

The eating out culture of the young adult population was regarded as normal and viewed as heavily influenced by ready availability and cost of food. It was suggested that for those who did not eat out regularly, it was unimportant whether the food was healthy or not. There was a perception that unhealthy food tasted better than healthy alternatives and could be an occasional reward or treat.

#### 3.1.2. Health Consequences of Food and Eating Out

Participants were mostly aware of some health consequences of making poor food choices. The link between diet and diabetes mellitus was raised with some citing personal experience with family members and the influence it had on meal choices when eating out. These young adults also raised issues such as weight gain and even inflammation being related to the consumption of unhealthy foods; however, for most participants these negative effects played a small role in their food choice. Messaging that emphasized the positive and immediate health effects of foods were viewed as effective messages rather than negative messaging with distant consequences.

### 3.2. Environmental Factors

#### 3.2.1. Smartphones as Influencers

This sub-theme had two major dimensions: the influence of continual smartphone-mediated access to social media in driving food choices and the use of the smartphone to instantly procure take-away food.

The use of social media to market foods online as well as individuals posting their own photos was viewed as a powerful driver of food selections among young adults. Images and good reviews were reported as supporting the choice of lesser known food outlets and cuisine types. Appealing pictures that looked good and captured the food in a positive way was enough to convince participants to order it. The participants further explored the use of imagery as a positive and negative force for influencing food choice. They suggested that more images of healthier food options would be a useful means of nudging young adults towards dietary improvements. Alternatively using ‘negative imagery’ might discourage the consumption of unhealthy foods. Participants recalled seeing images of some meals accompanied by the amount of fat in each dish. Another spoke about negative imagery portrayed in the media. Although the majority agreed such images would be a deterrent from consuming unhealthy take-away foods, they preferred positive over negative imagery as a means of encouraging healthy eating.

#### 3.2.2. Food Pricing and Discounts

There was some agreement that healthy food is expensive relative to unhealthy food and thereby becomes a barrier for young people to make adjustments to their eating out behavior. However, there was acknowledgement that health-conscious people were positively influencing the availability of healthy foods and that discounts on healthier food would be an incentive for young adults. Some participants said they would travel a further distance for eating out and take-away meals if the food was less expensive and represented value for money and, as noted above, this was easily facilitated by searching food websites on their smartphone.

#### 3.2.3. Menu Labelling Changes

Many participants suggested improvements to the current menu labelling available. They regarded the addition of kJ labelling an effective means of encouraging participants to choose healthier meals through lower energy. However, it was acknowledged that many people had no concept of what a kilojoule was and other suggestions such as listing the amount of added sugars or expressing the energy content of food in terms of the amount of physical activity required to balance the energy consumption would be more helpful to encourage healthy eating.

Inclusion of ingredient lists on menus was proposed as a method to allow informed choices on healthy foods and could be accompanied by cooking methods. They were aware that food preparation methods such as frying rather than grilling would alter the healthiness.

#### 3.2.4. Endorsements of Foods

Participants expressed trust in the endorsement of foods by credible organizations but not by restauranteurs or food outlets. It was commented that words such as ‘natural’ or ‘organic’ were used in menus to infer, ‘healthier options’ but they disbelieved such claims unless they came from a credible body.

The use of a simple and quickly comprehensible rating system endorsed by a credible organization was perceived as an effective means to provide and nudge young adults towards healthy choices when eating out. A system that enabled gradations of healthiness was preferred rather than a simple endorsement. The participants suggested a numerical scale or traffic light system would be practical for menus in terms of menu space and overhead menu boards as these could be easily placed beside each individual menu option.

However, it was not only the ratings and approval of credible organizations that shaped choice but also online reviews and endorsements by friends and other young adults were reported as having a large influence when eating out.

### 3.3. Minor Theme

A minor theme raised in one focus group by one participant concerned portion sizes and the role of large portions in overconsumption. Other groups did not explore this theme.

## 4. Discussion

The focus group participants articulated a number of actionable suggestions as to how the current food environment might be altered so as to enable healthier choices for meals and snacks prepared outside the home. The discussion of strategies and messaging for individual behavior change was less pronounced. The younger generation demonstrates greatest ownership of smartphones and their use in many aspects of their life. Unsurprisingly it was a vehicle for food choice with ability to instantly order food and for endorsements and restaurant reviews. Menu labelling also featured prominently and the role it could play in education and food selection. The social food environment featured alongside the physical features of the food environment as influencing food selection when young adults were eating out.

Young adults’ ownership of a smartphone is almost universal in the US and Australia. Smartphones appear to expose young adults to social environments that sway meal choice. Social media programs like Instagram subject them to food images that may overtly and covertly influence choices when eating away from home [16] and many ‘social influencers’ regularly post food images. Additionally, smartphones provide instantaneous access to food ordering systems that must now be considered a real part of the “away from home” food environment. A recent study sought to understand the food choices of 195,333 Deliveroo customers who ordered 1,613,968 meals or snacks over two months in 197 cities from 30,552 restaurants [17]. People engaged in sophisticated exploration but over time this practice lessened. It was found that people began to use their similarity-generalizations such as cuisine type or restaurant ratings to guide food choices. People who had a bad experience while exploring were less likely to be adventurous in future searching. Selecting a healthy food that was a bad experience resulted in a switch back to unhealthy food. The researchers suggested that to guide people towards further exploration of healthy choices categorizing these meals with another popular food category that was a good experience might result in further exploration of healthy foods available. Clearly, in this era of exploration of big data from apps, companies can develop machine learning and algorithms to influence food choice. Public health must address this in any measures to regulate the consumption of foods prepared outside the home.

Price discounts were important to the participants as might be expected given the limited budgets of full- or part-time students. Generally, results of studies testing discounts as incentives for the consumption of healthier foods are positive. Price discounts or voucher systems are most commonly employed and the setting has typically been supermarkets and worksite or university cafeterias [18]. Foods discounted are mostly fruits and vegetables but also healthier foods in general. The magnitude of the discount may be important but there has been little study of the dose-response effects. Discounts in the studies included in the cited systematic review ranged from 10% to 33% discount or vouchers ($1 to $22). Two studies targeted the food environments of university students offering discounts on fruit purchases (one simultaneously taxed French fries) and demonstrated increased purchases but durations were only six and ten weeks [19]. Two additional studies in university settings conducted during the 1990s showed increases in fruit and salad consumption with discounts in a cafeteria [20] and increases in low fat choices from vending machines when low fat snacks were reduced by 50% [21]. The issue of taxation of unhealthy foods did not arise during our focus groups, but previous research with young adults in Australia has indicated that they are less in favor of restrictive policies, e.g., taxation of sugar-sweetened beverages [22] and a Canadian survey of 16 to 30-year-olds also discovered there was less support for taxation of foods [23]. Thus, price discounts should be considered an option for improving the nutritional quality of foods consumed outside home.

The addition of menu labels to enable informed decisions and prompt healthier eating is well recognized. In the state in which this focus group sample resided, it is mandatory for outlets with more than 20 restaurants within the state (or 50 across the country) to include the number of kilojoules on menu boards and within written menus. This approach has been employed in other countries such as the US. The effectiveness of menu-labelling approaches was demonstrated in a systematic review reporting nine of 15 studies showed positive outcomes such as decreased energy consumption or ordering of lower energy options. Meta-analysis of the three studies measuring energy consumed (two in real world settings; 1938 participants overall) showed that the mean consumption of energy in the away from home food environment declined by 419.5 kJ 95% CI (−613.25, −225.76) (100.2 kcal) [24]. A more recent Cochrane review included three different randomized controlled trials of labelling versus no labelling in the restaurant setting and found a decrease in the energy of the food purchased of −46.72 kcals (−78.35, −15.10) among 625 participants [25].

A previous study in the university environment from which many of the participants in the current study were recruited found that only about 10% of students were aware of and used the energy labels when they were added to the menu and advertised with social marketing (*n* = 264) with 42% aware but not influenced. Those using the labels selected food with a mean of 978 kJ 95% CI (129.7, 1721.6) less than participants who did not [26]. This highlights that menu-labelling will only ever be used by some of the population and other research has indicated that there is a socioeconomic gradient in terms of those who notice and use the labelling. A statewide campaign in NSW, Australia in 2012 that introduced menu labelling to restaurants with 20 or more outlets in the state found the recall of the advertising for the program by 18 to 24-year-olds was around 40% with a mean reduction of 519 kJ per meal [27]. There seems to be considerable support for labelling among young adults in this University [26] and as evidenced in the Canadian study survey of 16 to 30-year-olds [23].

The young adults in this study suggested labels that gave an indication of the energy content in terms of physical activity required to offset the activity would be useful. A study in US college students compared exercise labels (minutes of brisk walking for equivalent energy) versus calorie labels versus with a no-label control [28] finding both forms of energy label resulted in significantly less energy ordered and consumed compared with the control. Thus, it appears there is not a specific need to change current energy-labelling.

While the evidence base for nutritional labelling is generally of a low quality no overall harmful effects are associated with labels although some researchers express concern about impacts on people with eating disorders. Thus, the current strategies of menu-labelling are supported. The main problems may be the reach of labelling given that a majority of food outlets and restaurants are independent and thus fall outside the current legislation for labelling and that investment is needed in raising awareness of labelling via a social marketing campaign.

Endorsements from credible sources and friends were viewed as positively influencing food selection. Previously a credible source has been identified as an effective behavior change technique [29]. Peer-influences are strongest during the adolescent years and can continue during early adulthood, so these findings are not unexpected. Food endorsement schemes from a credible source like government or non-government health organizations may facilitate healthier choices when eating outside the home. Suggestions for rating of foods on a scale rather than an all-or-none approach have gained momentum in Australia via the Health Star Rating system (HSR) applied to packaged foods which can range from 0.5 stars to 5 stars (most healthy) [30]. The HSR scheme was designed to compare packaged foods within a category but research to examine the comparison of fast foods with packaged foods when the HSR was calculated found a similar spread of stars with a mean of 2.5 stars for fast foods and 2.6 for packaged [31]. The HSR is currently being reviewed in Australia in order to address identified shortcomings in classifications of some foods. The rating addresses nutritional quality and amounts of nutrients of public health concern in a food (saturated fat and sodium). The effectiveness of the HSR compared with kilojoule labelling to improve fast food selection under simulated conditions was tested in a four-group experiment with no label, kilojoule label, HSR label and kilojoule label with HSR. It might be expected that adding HSR would improve overall nutritional profile but the kilojoule label with HSR actually resulted in food with a poorer nutritional profile than KJ alone [32]. Other food labelling systems with a gradation includes the traffic light system, red, amber and green. However, comparison of no label with energy or single color of traffic light failed to have any influence on parents’ selection of foods for their children in an experimental setting [33].

Only two themes arose around personal behavior during the focus groups. It is of interest that participants viewed eating out as a treat so that the food choice did not matter. However, the selection criteria for the group included eating out at least twice weekly, a level that has been associated with harmful effects in young adults [6,34]. Our quantitative study has demonstrated one in four meals and snacks are prepared outside the home so clearly some guidance on what constitutes frequent eating out will be required. Secondly, we found the subjects were unconcerned about food choices and health when eating out although they knew possible disease consequences of poor food choice such as type 2 diabetes mellitus. Typically, the young perceive the outcome of poor lifestyles as so distant it is of no immediate concern [35]. The issue of large portion sizes was only raised during one focus group. Portion sizes of fast foods have increased over the past decade and warrants further investigation to improve food environments for healthy eating [36].

There are some limitations of the current study with respect to the sample recruited for the focus groups. While we recruited a sufficient number of participants for data theme saturation there were more than twice as many females as males participating which is common for food-related studies. Despite our best efforts to recruit from suburbs with lower socioeconomic status, the sample consisted of 19 participants from the two most advantaged socioeconomic groups. This raises issues around the generalizability of the findings and these findings might only be applicable to similar groups.

## 5. Conclusions

In conclusion, these 18 to 30-year-olds appeared to be unconcerned about their own personal behaviors around eating out and nutritional value of their choices. However, they identified a range of food environment and social environment-related factors that would enable healthier choices when eating meals prepared outside the home. Some of these such as price discounts and food labels have already been shown to have positive outcomes on diet. They also specified the continuing influence of peers in decision making around food and pinpointed growing influences such as smartphone apps for ordering of food and accessing reviews and persuasive food imagery posted by friends and others on social media. These later influences warrant further attention. It is suggested that government agencies continue to develop menu labelling systems and extend their reach to allow informed decision-making by the public. Any social marketing campaign needs to raise awareness that eating out twice weekly or more is associated with weight gain. Changes in food pricing deserve further discussion with food industries and government.

## Figures and Tables

**Table 1:** Questions used to guide the focus group discussions.

Where do you like to eat?
Where do you eat out most?
When eating out, how do you choose where to eat?
What reasons would make you choose (café, restaurant, take-away, fast food chain)?
If you are in a (café, restaurant, take-away, fast food chain) how do you choose the foods and drinks you eat?
When you’re eating out, do health factors influence your choices?
If yes, what health related factors influence your choices?
When you’re eating out, what, if anything, influences you to make healthier food and drink choices?
What would encourage you to choose healthier options?

**Table 2:** Illustrative quotations of themes and sub-themes from the young adult participants.

Themes	Sub-Themes	Illustrative Quotations
Personal behaviors	Indulgence when eating out	I think of taste as well in terms of richness and I think health comes in to that. I guess I cook pretty healthily when I am cooking for myself, so when I am eating out I tend to indulge more, so I am not really worried about necessarily how healthy the food is. (Male) It also depends, it’s potentially not something that you would do all the time and so that could be something you treat yourself to, you are thinking this is significantly unhealthier but it’s the only unhealthy thing I’ll eat in a week so it’s kind of alright. (Female)
	Health consequences of food and eating out	If I’m eating out with someone I care about who has health issues then I’m not going to take them to KFC or McDonalds, like I’m not going to put them in a situation where they’ll be tempted. (Female) I think it’s important that when you say something, messages shouldn’t be shaming the person. For example, if you say, this dish has no serves of vegetables in it, and you say something like, eating more carrots improves your eyesight. Something positive. I don’t think people react very well to negative messages. (Female)
Environmental factors	Smart phones as influencers	I also look at the ratings but also just look on Instagram they post photos of nice food, I’ll search it up and I’ll probably go there. (Female) I’m vegetarian I feel like there are not many options in fast food so we use a lot of different apps and see what deals they might have or what kind of food I feel like on the day, a specific cuisine each time. (Female) I’ve been trying different cuisine and different foods, there are a lot of apps and websites offering discounts and promotions I’ve been using that lately, typically I’ve been eating a lot of Chinese food, Thai, Korean, Jap with the app… (Female)
	Food pricing and discounts	That’s another big thing with the choice to eat healthy is again the cost of the food, the healthier option always seem to be more expensive because they’re fresh ingredients rather than just deep fried. (Male) If there’s a deal, I might go there again, I might go an extra mile to go there as well. (Male) I suggest when I order lower sugar drink there will be some discount, maybe 20 cent discount or something. (Female)
	Menu-labelling	I think if they had the calories next to the item, it would stop me from ordering a lot of them. (Female) There’s KJ next to the menu item, in fast food, it’s unhealthy but I choose the one that’s less. (Male) I think knowing the tablespoons of sugar, if it says energy I wouldn’t know what it is, 10 teaspoons of sugar I understand that, it’s a lot. (Male) I think it is better to advertise you know how many exercises you have to do to burn that food beyond the restaurant, like in a gym or something, so you get the information but you are not as put off, but you still have that self-conscious about eating out and what you choose. And say for example it says one hour on the treadmill, rather than doing that, say for example, if you consume a coke you have to do 50 burpies. (Female) List the ingredients as well. Some menus don’t have the ingredients, just the title of the dish or a vague description. If it really outlines what the ingredients are you can get a better sense of what you are having and decide if they are healthy ingredients or not. (Male) I think how menus are written, would be helpful so sometimes the new cafes or brunch places list out their ingredients that are on there, but often times it’s just a nice name: like lasagna and you don’t know what’s in it. (Female)
	Endorsements	Even if I don’t believe the natural or organic part if it says this organization recommends it I wouldn’t research it, I’d just believe it. (Male) I don’t trust advertisements by default, I don’t agree with words like organic, natural. Something in me just thinks that sort of stuff is for a different type of person. The things I trust is like a news article, things that present themselves as news articles, reputable documentation, something that looks like a credible source. (Male) Sometimes back home they have the health check or something beside the item on the menu, like the Heart Foundation tick, like that means that dish is healthy if I went to a restaurant and was looking for a healthy dish that sign would indicate it. (Female) If we’re going to a brand new restaurant that no one has ever heard of what we do is, we use Google reviews to see what people have ordered then you base your order off what they ordered and you sort of go from there. (Female) I look for highly rated restaurants because I want to maximize my money. So I make sure that I go there and order and I’ll be happy after and not be disappointed. Maybe for other factors like it’s crowded or bad service, but if the food is good it’s fine. So the first thing I do is look for top rated cheap restaurants. (Female)
